# Response to "Evaluation of the injuries in earthquake victims with computed tomography"

**DOI:** 10.1590/1806-9282.20240285

**Published:** 2024-08-16

**Authors:** Gokhan Tonkaz, Demet Sengul, Tumay Bekci, Ilker Sengul, Ismet Mirac Cakir, Esma Cinar, Duygu Erkal Tonkaz, Tugrul Kesicioglu, Iskender Aksoy, Serdar Aslan

**Affiliations:** 1 Giresun University, Faculty of Medicine, Department of Radiology - Giresun, Turkey.; 2 Giresun University, Faculty of Medicine, Department of Pathology - Giresun, Turkey.; 3 Giresun University, Faculty of Medicine, Division of Endocrine Surgery - Giresun, Turkey.; 4 Giresun University, Faculty of Medicine, Department of General Surgery - Giresun, Turkey.; 5 Gumushane State Hospital, Department of Radiology - Gümüşhane, Turkey.; 6 Giresun University, Faculty of Medicine, Department of Emergency Medicine - Giresun, Turkey.

Dear Editor,

We read with a great deal of interest the article "Evaluation of the injuries in earthquake victims with computed tomography," published in *Revista da Associação Médica Brasileira*, Volume 70^1^. We appreciate the authors’ interest in our work and would like to respond and address some of the issues raised by the authors and dispel some possible misconceptions about our work. The authors considered the major limitation of our study to be its dependence on computed tomography (CT) scans alone, which may not always provide complete information in all cases^1^. Indisputably, it would be helpful to expand the scope of the study by including a larger sample size and more diagnostic tools as the authors have mentioned. However, we have already stated that our article solely investigated CT imaging characteristics of earthquake victims after those catastrophic disasters, such as the 2023 Turkey-Syria earthquakes, by using the Teleradiology Reporting System (TRS) of the Ministry of Health, Republic of Turkey^2^. For this purpose, we retrospectively analyzed hospital data from a total of 11 cities affected by the earthquake in Turkey and presented the CT imaging characteristics of adult and pediatric earthquake survivors even in two separate studies, parts I and II, recently published in *Revista da Associação Médica Brasileira*, Volume 69^2,3^. Our study is on the "imaging" but not the laboratory characteristics of the victims of the aforementioned catastrophic great disasters, and we would like to emphasize the awareness of the situation that the authors have criticized in their letter. Therefore, in the "limitations" section of our study, we already included (i) not possessing trauma score data or patient outcomes such as mortality as a retrospective study, (ii) involving just the cases with CT images, and (iii) excluding the victims not undergoing CT scans, but diagnosing by imaging strategies such as direct radiography, sonography, or magnetic resonance imaging. We kindly like to express and render again that our original preliminary work is a needful, unique, and in-place evaluation of these great disasters right just 1 month after they happened, conversely being a kind of follow-up study^3^. In conclusion, our study is a revealing work in that the frequency of earthquake-related injuries varies according to different regions based solely on CT imaging by utilizing TRS, Turkey. We hope that the outcomes of our preliminary work might be beneficial in the development of relevant guidelines and disaster preparedness globally, particularly for future undesirable and unwelcome earthquakes.
